# Alterations of peripheral nerve excitability in an experimental autoimmune encephalomyelitis mouse model for multiple sclerosis

**DOI:** 10.1186/s12974-020-01936-9

**Published:** 2020-09-07

**Authors:** Nathalia Bernardes Teixeira, Gisele Picolo, Aline Carolina Giardini, Fawzi Boumezbeur, Géraldine Pottier, Bertrand Kuhnast, Denis Servent, Evelyne Benoit

**Affiliations:** 1 Université Paris-Saclay, CEA, Département Médicaments et Technologies pour la Santé (DMTS), Service d’Ingénierie Moléculaire pour la Santé (SIMoS), ERL CNRS 9004, Gif-sur-Yvette, France; 2 Laboratory of Pain and Signaling, Butantan Institute, São Paulo, Brazil; 3 Université Paris-Saclay, CEA, NeuroSpin, Gif-sur-Yvette, France; 4 Université Paris-Saclay, CEA, Inserm, BioMaps, Orsay, France

**Keywords:** Electrophysiology, Experimental autoimmune encephalomyelitis, Mouse, Multiple sclerosis, Myelin oligodendrocyte glycoprotein, Neuromuscular junction, Peripheral nervous system

## Abstract

**Background:**

Experimental autoimmune encephalomyelitis (EAE) is the most commonly used and clinically relevant murine model for human multiple sclerosis (MS), a demyelinating autoimmune disease characterized by mononuclear cell infiltration into the central nervous system (CNS). The aim of the present study was to appraise the alterations, poorly documented in the literature, which may occur at the peripheral nervous system (PNS) level.

**Methods:**

To this purpose, a multiple evaluation of peripheral nerve excitability was undertaken, by means of a minimally invasive electrophysiological method, in EAE mice immunized with the myelin oligodendrocyte glycoprotein (MOG) 35-55 peptide, an experimental model for MS that reproduces, in animals, the anatomical and behavioral alterations observed in humans with MS, including CNS inflammation, demyelination of neurons, and motor abnormalities. Additionally, the myelin sheath thickness of mouse sciatic nerves was evaluated using transmission electronic microscopy.

**Results:**

As expected, the mean clinical score of mice, daily determined to describe the symptoms associated to the EAE progression, increased within about 18 days after immunization for EAE mice while it remained null for all control animals. The multiple evaluation of peripheral nerve excitability, performed in vivo 2 and 4 weeks after immunization, reveals that the main modifications of EAE mice, compared to control animals, are a decrease of the maximal compound action potential (CAP) amplitude and of the stimulation intensity necessary to generate a CAP with a 50% maximum amplitude. In addition, and in contrast to control mice, at least 2 CAPs were recorded following a single stimulation in EAE animals, reflecting various populations of sensory and motor nerve fibers having different CAP conduction speeds, as expected if a demyelinating process occurred in the PNS of these animals. In contrast, single CAPs were always recorded from the sensory and motor nerve fibers of control mice having more homogeneous CAP conduction speeds. Finally, the myelin sheath thickness of sciatic nerves of EAE mice was decreased 4 weeks after immunization when compared to control animals.

**Conclusions:**

In conclusion, the loss of immunological self-tolerance to MOG in EAE mice or in MS patients may not be only attributed to the restricted expression of this antigen in the immunologically privileged environment of the CNS but also of the PNS.

## Introduction

Multiple sclerosis (MS) is a chronic, inflammatory, and demyelinating disorder of the central nervous system (CNS), considered as an important and frequent neurological impairment condition [1, 2]. It is a disorder of autoimmune origin, where the immune system recognizes parts of the CNS as antigens, specifically peptides that form the myelin sheath of axons of neurons [3–5] leading to a demyelination process which induces serious physical, cognitive, emotional, and social problems [1, 2]. Although the evolution of the disease is highly variable, the disability faced by most people is irreversible. Therefore, MS is considered incurable, and the different therapeutic options focus on delaying disease progression and promoting the relief of symptoms so as to maintain the quality of life of patients [2]. In contrast to therapies focused on controlling or modulating the immune (innate and adaptive) responses to limit demyelination and neuronal damage, novel drugs currently in clinical trial have been recently reported to promote repair and regeneration in the CNS [6].

The immunopathogenesis of MS is not completely understood, and it sets up as a picture that remains to be elucidated. However, important steps are clearly involved in this disease, primarily at the CNS. Both innate and adaptive immune system responses are dysregulated in MS [7]. It is a mainly T cell-mediated disease with a central role of myelin-reactive CD4^+^ T cells [8, 9]. Autoreactive T-helper type 1 (Th1) and 17 (Th17) cells are peripherally activated and subsequently migrate to the CNS, causing central inflammation with release of cytokines, microglial activation, axonal and myelin injury, followed by demyelination and atrophy of white matter tract across the brain and spinal cord [10–12], which causes neurological and motor impairment. The atrophy occurs in key regions. Posterior cingulate cortex, precuneus, and thalamus are among the earliest regions to become atrophic [13]. In addition to the neuroinflammation, some other factors may underlie the neurodegeneration and brain atrophy, including mitochondrial failure, iron deposition, and retrograde neurodegeneration in the deep gray matter through white matter lesions [14–16].

B cells and antibodies have also a role in the pathology of MS. High levels of immunoglobulins in the cerebrospinal fluid of patients were detected together with an increase in these levels during periods where the symptoms were worse [17–19]. The production of myelin-specific antibodies (and the consequent rupture of myelin sheets) seems to be an important way in which B cells contribute to the disease [19]. Recently, a more central role of B cells in MS, which appears to be antibody independent, has been described [20, 21]. According to this recent scenario, B cells would activate or downregulate the proinflammatory responses of both myeloid and T cells, and recruit autoreactive T cells to the CNS. Thereafter, interactions among these cells would determine the development of MS episodes.

In contrast to the well-defined and critical central effect, the peripheral alterations, although frequently described in patients, have not been characterized as thoroughly [22–25]. In particular, histological studies performed on rats with experimental autoimmune encephalomyelitis (EAE) showed that inflammation was present in both peripheral nervous system (PNS) and CNS [22]. In this model of EAE induced by passive transfer of a cytotoxic CD4^+^ T cell clone specific for the 72-89 peptide of guinea-pig myelin basic protein (MBP), the spinal roots in the PNS as well as the spinal cord root entry and exit zones in the CNS were considered as the main sites of demyelination at periphery. More recently, electrophysiological analysis revealed a membrane hyperexcitability of sensory neurons of dorsal root ganglia isolated from MOG_35-55_-induced EAE mice, providing evidence of peripheral sensitization in MOG-EAE murine model [23].

The frequency of peripheral demyelination in a whole MS population is unknown, although some case reports have been described to be associated with demyelinating neuropathy [26, 27].

Few studies have investigated peripheral alterations induced by MS. Hence, peripheral sensory and motor abnormalities were analyzed in 20 patients showing MS by evaluating conduction velocities and amplitudes of ulnar, sural, and tibial nerves. Electrophysiological abnormalities were found in 15 of 91 nerves examined (16.5%) but neurological disability was not associated with the presence of electrophysiological abnormalities [28]. In addition, nerve conduction abnormalities suggestive of demyelination were demonstrated in only 5% of 60 patients with relapsing-remitting MS [29]. This later study, in contrast to a number of case reports describing patients with MS who develop demyelinating neuropathy, strongly suggests that central and peripheral demyelination coexist only in a special subgroup of patients with MS.

The aim of the present work was to study the peripheral impairment induced by MS. To this purpose, peripheral sensory and motor nerve excitability was evaluated in a MOG_35-55_-induced EAE mouse, an experimental model for MS that reproduces, in animals, the anatomical and behavioral alterations observed in humans with MS, including CNS inflammation, demyelination of neurons, and motor abnormalities [3, 30–32].

## Materials and methods

### Animals

Animal experiments are reported in line with the ARRIVE (Animal Research: Reporting of In Vivo Experiments) guidelines developed in consultation with the scientific community as part of an NC3Rs initiative to improve standards of reporting the results of animal experiments, maximizing information published, and minimizing unnecessary studies [33, 34]. Experiments were carried out on 8-week-old female C57BL/6 mice (*Mus musculus*, weighting 18–20 g) purchased from Janvier Elevage (Le Genest-Saint-Isle, France). We used exclusively female mice taking into account that first, MS is an autoimmune disease where the incidence is higher in women than in men [35–37] and second, most of the previous studies were performed on female animals. The animals were acclimatized at the CEA animal facility for at least 48 h before experiments, and were treated in strict adherence with the European Community guidelines for laboratory animal handling and to the guidelines established by the French Council on animal care “Guide for the Care and Use of Laboratory Animals” (EEC86/609 Council Directive—Decree 2001-131). In particular, the mice were housed in a room with controlled temperature and a 12-h light/12-h darkness cycle, in standard laboratory cages with environmental enrichment (bedding and cardboard tubes), and were allowed to free access to water and food.

All animal experimental procedures were approved by the Animal Ethics Committee of the CEA, by the French General Directorate for Research and Innovation (project APAFIS#5973-2016070515456532v6 authorized to FB and project APAFIS#2671-2015110915123958v4 authorized to EB) and by the Butantan Institute (CEUAIB protocol number 7334170718 authorized to GP).

### EAE mouse model

EAE was induced as previously described [38, 39]. Briefly, each EAE mouse was immunized with 200 μg of synthetic myelin oligodendrocyte glycoprotein (MOG) 35-55 peptide (MEVGWYRSPFSRVVHLYRNGK) with purity greater than 95%. The peptide was emulsified in incomplete Freund’s adjuvant (IFA; InvivoGen, France) supplemented with 400 mg/mL of *Mycobacterium tuberculosis* to lead to complete Freund’s adjuvant (CFA), and injected subcutaneously near the base of the tail in a 200-μL volume. Immediately after immunization and 2 days later, mice were intraperitoneally injected with 300 ng/kg of pertussis toxin from *Bordetella pertussis* bacteria (PTX; Sigma-Aldrich, Saint-Quentin Fallavier, France) in a 200-μL volume. The control animals (CFA-mice) were similarly injected with CFA free of MOG_35-55_ peptide and pertussis toxin.

Six mice (MOG-M1 to MOG-M6) were immunized at day 0. Recordings were performed at days 14 and 28, i.e., 2 and 4 weeks after immunization, and compared to those of six age-matched control animals (CFA-M1 to CFA-M6). All mice were daily observed for clinical signs. The references used to assess the clinical scores of EAE mice, assigned by an observer who was blinded to the treatment, were the flaccidity or paralysis of the tail (first sign), and the drag of the hip without limb paralysis (second sign) or associated with hind-limb (third sign) and then fore-limb (forth sign) paralysis.

### In vivo electrophysiology

Recordings were performed by means of a minimally invasive electrophysiological method. The principle is to electrically stimulate a nerve trunk and to record, in return, the compound action potential (CAP) resulting from the activity of all fibers composing the stimulated nerve (“sensory nerve recordings”) or muscle (“motor nerve recordings”).

**Fig. 1:**
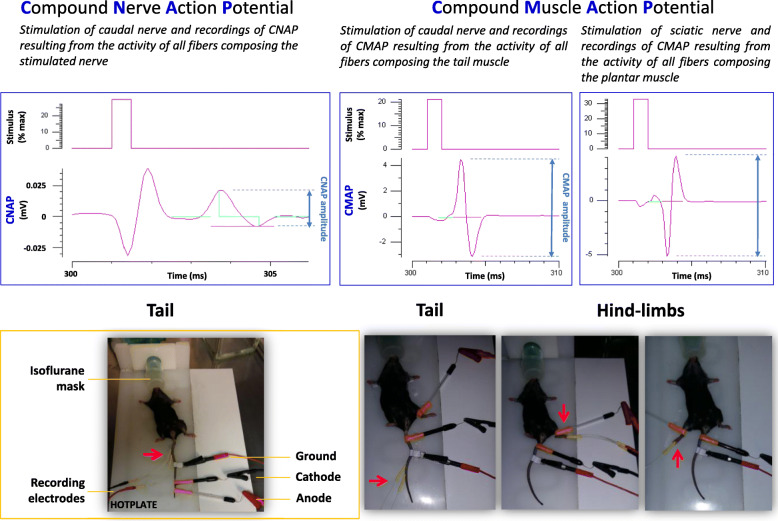
Traces of compound nerve action potential (CNAP) and compound muscle action potential (CMAP) for sensory and motor nerve recordings, respectively, evoked by nerve stimulation (upper), and corresponding locations of stimulation and detection (red arrows) electrodes (lower)

After being weighed, each mouse was placed in an anesthesia-induction chamber in which a mixture of oxygen (0.4 L/min via an oxygen extractor), air (0.2 L/min via an air condenser), and isoflurane (AErrane®, Baxter S.A., Lessines, Belgium; 2.0–2.5% via an anesthetic diffuser) was diffused. When the mouse was asleep, it was taken out of the chamber and set on a plate heated using water circulation (via a T/PUMP). Its muzzle was positioned at the level of a mask which continuously delivered the anesthetic gas mixture to keep the animal asleep, and its two hind-limbs were fixed by adhesive tape (Fig. 1). If necessary, the percentage of isoflurane was adjusted to maintain the anesthesia. The animal temperature was measured using a digital thermometer equipped with a rectal probe.

The electrical stimulations were delivered to either the caudal or sciatic nerve by two stimulators (A395, World Precision Instruments, Sarasota, FL, USA) via two non-polarizable Ag/AgCl external electrodes, the anode (RC4, World Precision Instruments) and the cathode (RC3, World Precision Instruments), directly affixed to the skin of the mouse. A medical gel (Polaris II) was used to improve the contact, i.e., the electrical conduction, between the electrodes and the skin. For sensory nerve recordings, the electrodes were located at the distal portion of the mouse tail, the anode being in the most distal position and the cathode about 1 cm from the anode. For motor nerve recordings, the anode was placed at the level of the ankle of the right or left hind-limb studied, and the cathode at the base of the tail (Fig. 1).

The CAP [compound nerve action potential (CNAP) and compound muscle action potential (CMAP) for sensory and motor nerve recordings, respectively], which propagated in the stimulated nerve, was collected by means of two detection electrodes E1 and E2 (MF3.OE.1F35.12, Comepa) which were very fine needles inserted (i) in the proximal part of the tail, the electrode E2 being in the most proximal position and the electrode E1 about 1 cm from the electrode E2 (sensory nerve recordings); (ii) in the distal part of the tail, the electrode E2 being in the most distal position and the electrode E1 about 1 cm from the electrode E2 (motor nerve recordings from the tail muscle); and (iii) in the right or left hind-limb (motor nerve recordings from the plantar muscle). These electrodes were connected to an amplifier (Disa EMG 14C13, Sklovlunde) to increase the CAP amplitude, and then to a “hum bug” (Quest Scientific) to eliminate the sinusoidal noises that are inherent to electrophysiological recordings. Finally, a ground electrode was placed between the cathode and the detection electrodes for sensory and motor nerve recordings from the tail, or below the cathode for motor nerve recordings from the right or left hind-limb (Fig. 1).

The multiple evaluation of sensory and motor nerve excitability properties was performed using the Qtrac© software (H. Bostock, Institute of Neurology, London, U.K.). By means of a digital-to-analog converter (DAQ2000, Iotech), this software allowed the delivery of the stimulation sequences and, in return, managed the recordings (at a sampling frequency of 10 kHz) and analysis of the CNAP and CMAP collected from the stimulated nerve and muscle, respectively (Fig. 1). It should be emphasized that, under these conditions, the CAP in response to a single stimulation was biphasic, i.e., a positive phase followed by a negative one or a negative phase followed by a positive one, according to the relative positions of the two electrodes, since it represented the potential difference between the two detection electrodes, i.e., E1-E2. Its amplitude was measured as the absolute difference between the maxima of these two phases (Fig. 1).

### Protocols, data analyses, and statistics

**Fig. 2:**
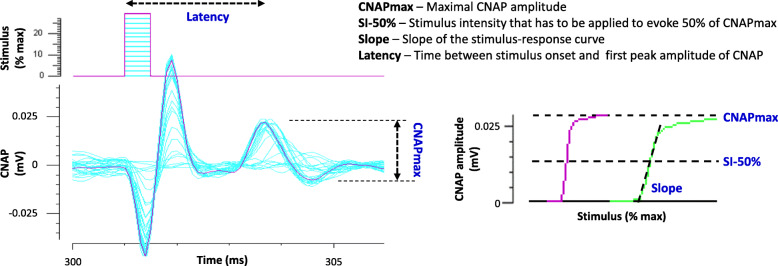
Stimulus-response curves (i.e., CNAP in response to nerve stimulations of increasing intensity) and derived excitability parameters (left). Relationships between the CNAP amplitude and the stimulation intensity delivered manually (in pink) and then automatically (in green) by the program (right)

The stimulation protocol (“QTracS” program) lasted a few minutes and consisted in establishing the stimulus-response curve, i.e., the relationship between the CAP amplitude and the stimulation intensity, as exemplified for the CNAP in Fig. 2. Firstly, the CAP amplitude was measured as a function of the intensity of the stimulation (electric current) of 1-ms duration which, starting from 0, was gradually increased by steps of 3% of its maximum value (i.e., 2 mA, each of the two stimulators delivering a maximum of 1 mA), and manually until a maximum CAP amplitude was obtained. Secondly, a stimulus-response curve was generated automatically by the program, from which four parameters characteristic of sensory nerve or neuromuscular excitability were estimated (“QTracP” program). These parameters included (1) the maximal CAP amplitude (CAPmax), which depended on the number of muscle and nerve fibers responding to stimulation; (2) the stimulation intensity necessary to generate a CAP with an amplitude equal to 50% of its maximum value (SI-50%), which depended on the excitability threshold of fibers; (3) the slope of the stimulus-response curve (Slope), which depended essentially on the passive membrane properties of fibers; and (4) the time between the stimulus onset and the first peak amplitude of CAP (Latency), which depended on the CAP propagation/transmission velocity. Therefore, changes in these parameters gave information mainly on the density and functional state (activation) of voltage-gated sodium (Na_V_) and potassium (K_V_) channels, as well as on the passive membrane properties of nerve fibers linked, in particular, to the presence or absence of a myelin sheath surrounding the axons.

Data are presented as means ± standard deviations (S.D.) of at least 5 (n) different mice (6 and 5–6 animals 2 and 4 weeks after immunization, respectively). Differences between values were tested using the parametric two-tailed Student’s *t* test (either paired samples for comparison within a single population or unpaired samples for comparison between two independent populations), and the one- or two-way analysis of variance (ANOVA for comparison between the means of independent populations) or the non-parametric Mann-Whitney *U* test, depending on the equality of variances estimated using the Lilliefors’ test. Differences between results are considered to be statistically significant for a *P* value of less than or equal to 0.05.

### Evaluation of myelin thickness through transmission electronic microscopy

Under anesthesia with ketamine and xylazine (75 mg/kg and 10 mg/kg, respectively, intraperitoneal), mice were perfused with modified Karnovsky fixative solution containing 2.5% glutaraldehyde and 2% paraformaldehyde in 0.1 M sodium phosphate buffer solution (pH 7.4). The muscles of the right hind leg were dissected and the right sciatic nerve was collected. Samples were post-fixed in a solution of 1% osmium tethoxide, at 4 °C, followed by immersion in a 5% aqueous uranyl acetate solution at room temperature. After dehydration in alcohol, samples were immersed in propylene oxide and then included in Spur resin. Samples were sectioned (semi-thin sections—15 μM) in an ultra-microtome (Reichert Ultra Cut®) and stained with 1% toluidine blue solution. Subsequently, ultra-thin sections were cut (60 nm), collected them on 200 mesh copper grid (Sigma®), and the contrast was obtained using 4% uranyl acetate solution and 0.4% aqueous lead citrate solution [40]. The grids were examined in the Jeol 1010 transmission electron microscope (Department of Anatomy at the University of São Paulo) and quantification was performed using ImageJ software (NIH/EUA).

## Results

### Clinical scores of EAE and control mice

**Fig. 3:**
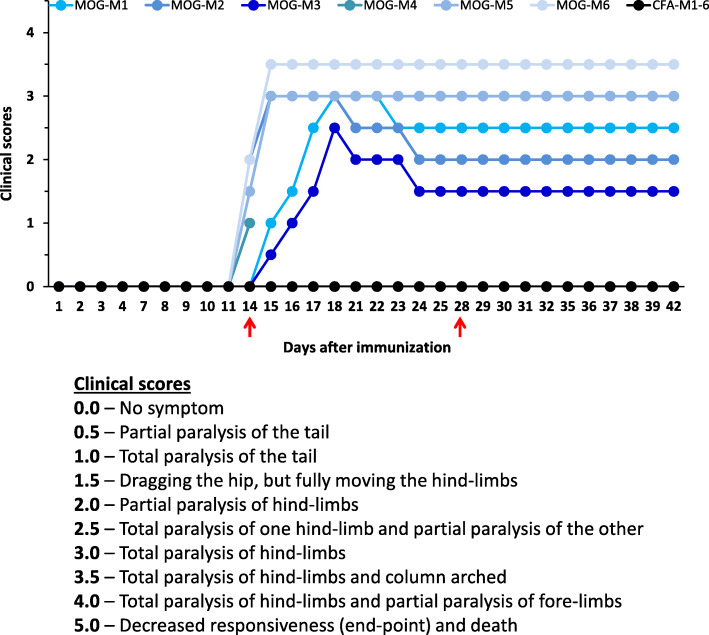
Individual clinical scores of 6 EAE mice (MOG-M1 to MOG-6) and control mice (CFA-M1 to CFA-6) as a function of time after immunization at day 0. The red arrows indicate the days of electrophysiological recordings (i.e., 2 and 4 weeks) after immunization

The clinical scores of MOG- and CFA-mice were determined every day after immunization, for 6 weeks, to describe the motor symptoms that occurred in the progression of EAE (Fig. 3).

As expected, the mean clinical score increased from 0.0 to more than 2.5 within about 18 days after immunization for MOG-mice while it remained null for all CFA-mice. It is worth noting that a quite large clinical score variability occurred between EAE mice.

### Mouse body weight and temperature

**Fig. 4:**
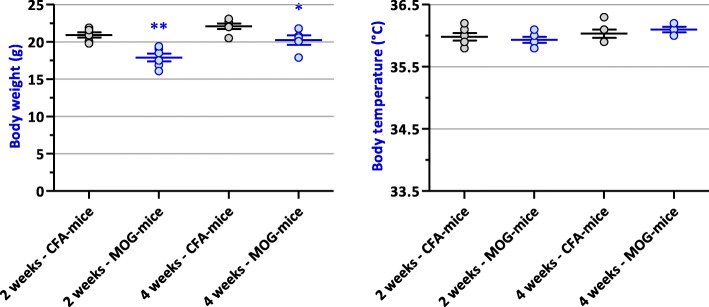
Body weight (left) and temperature (right) of EAE (MOG) mice, compared to control (CFA) animals, 2 (*n* = 6) and 4 (*n* = 5–6) after immunization. *P = 0.027 and **P = 0.001

Significant decreased body weight were observed in MOG-mice compared to CFA-animals, 2 weeks (*P* = 0.001) and, although less pronounced, 4 weeks (*P* = 0.027) after immunization (Fig. 4). However, compared to initial body weight measurements performed the day of immunization (day 0), i.e., 19.58 ± 0.43 (*n* = 6), EAE mice lost weight 2 weeks after immunization (*P* = 0.0002) and then gained weight 4 weeks after immunization (*P* = 0.435). On the contrary, control animals homogeneously gained weight (*P* < 0.013), compared to initial body weight measurements performed at day 0, i.e., 19.63 ± 0.28 (*n* = 6).

No significant change (*P* ≥ 0.492) in body temperature was detected between EAE and control mice, whatever the number of weeks after immunization (Fig. 4).

### Sensory nerve excitability properties (CNAP recordings)

**Fig. 5:**
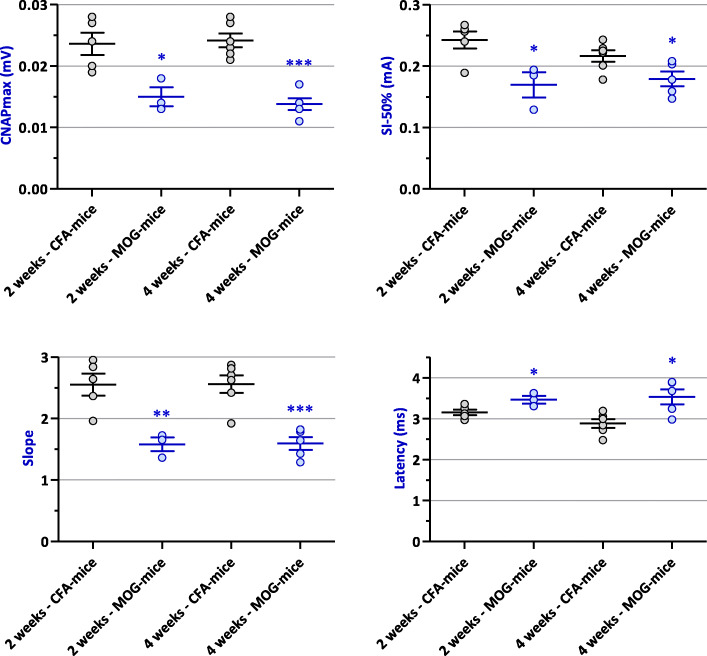
Maximal CNAP amplitude (CNAPmax), stimulation intensity necessary to generate a CNAP with an amplitude equal to 50% of its maximum value (SI-50%), slope of the stimulus-response curve (Slope), and time between the stimulus onset and the first peak amplitude of CNAP (Latency) in EAE (MOG) mice, compared to control (CFA) animals, 2 (*n* = 3–5) and 4 (*n* = 5–6) weeks after immunization. *P ≤ 0.035, **P ≤ 0.008, and ***P ≤ 0.0005

As shown in Fig. 5, a significant decrease of the maximal CNAP amplitude (*P* ≤ 0.012) and of the slope of the stimulus-response curve (*P* ≤ 0.008), as well as a significant increase in the time between the stimulus onset and the first peak amplitude of CNAP (*P* ≤ 0.035), were observed in MOG-mice, compared to CFA-animals, at any given time-point. This indicates a lower propagation velocity of CNAP in EAE than in control animals.

**Fig. 6:**
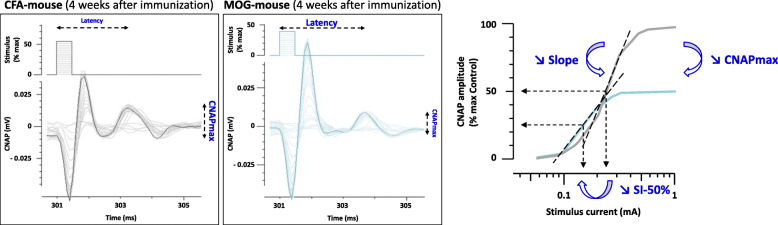
Example of increased Latency and decreased CNAPmax, Slope, and SI-50% in an EAE (MOG) mouse, compared to a control (CFA) animal, 4 weeks after immunization

These changes in the four parameters derived from the stimulus-response curve are exemplified in Fig. 6 by CNAP recordings obtained from individual MOG- and CFA-mice, 4 weeks after immunization.

**Fig. 7:**
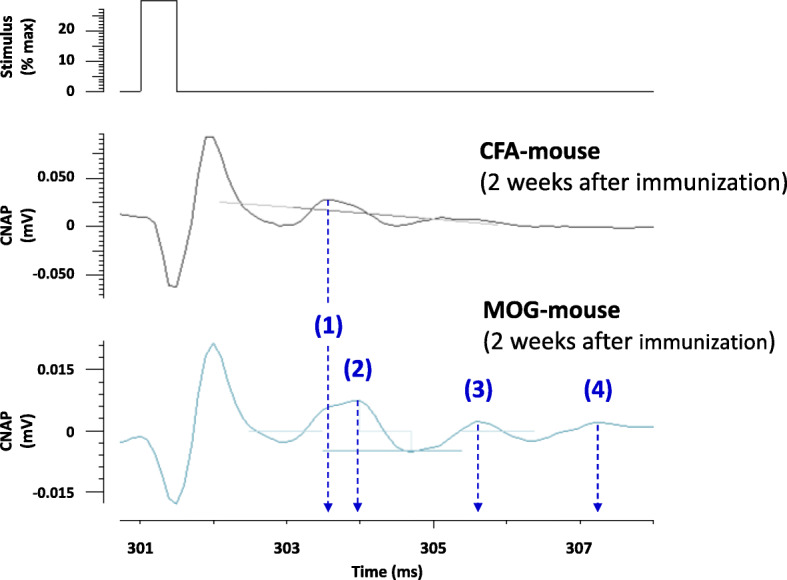
Low propagation velocity of CNAP (increased Latency) and sensory nerve hyperexcitability (decreased SI-50%) in EAE (MOG) mice, compared to control (CFA) animals, are exemplified by CNAP recordings showing (1) “control-like” (i.e., unaffected), (2) “slower”, (3) “very slower”, and (4) “very very slower” conducting nerve fibers in a MOG-mouse, compared to a CFA-animal, 2 weeks after immunization

Similarly, low propagation velocity of CNAP (increased Latency) and sensory nerve hyperexcitability (decreased SI-50%) in EAE mice, compared to control animals, are exemplified in Fig. 7 by CNAP recordings obtained from individual MOG- and CFA-mice, 2 weeks after immunization.

### Motor nerve excitability properties (CMAP recordings)

**Fig. 8:**
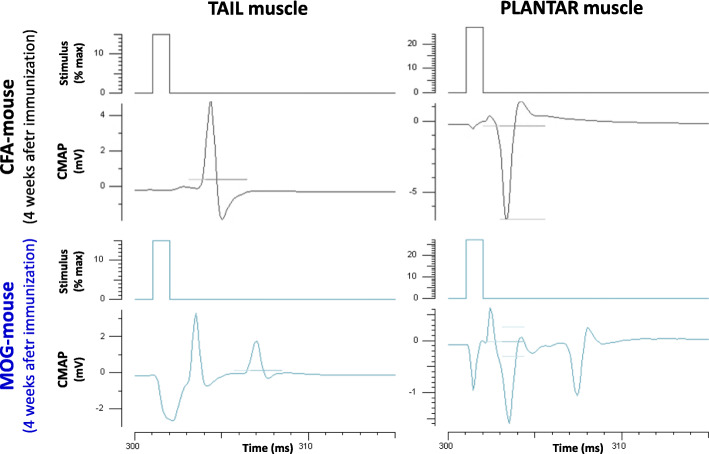
Traces of CMAP recorded from the tail and plantar muscles of a control (CFA) mouse and an EAE (MOG) animal, 4 weeks after immunization

The CMAP recorded at the tail and plantar muscles of EAE mice was generally followed by a second CMAP (of reduced amplitude), as illustrated in Fig. 8, 4 weeks after immunization.

The second CMAP was detected in 50% EAE mice (3/6 animals) 2 weeks after immunization while, 4 weeks after immunization, it was systematically observed in 100% EAE mice (5/5 animals). This second CMAP never occurred in control mice (6/6 and 6/6 animals, 2 and 4 weeks after immunization, respectively). As already stated in the “Materials and methods” section, the CMAP in response to a single stimulation consisted of a positive phase followed by a negative one (as exemplified by tail muscle recordings) or a negative phase followed by a positive one (as exemplified by plantar muscle recordings), according to the relative positions of the two detection electrodes.

**Fig. 9:**
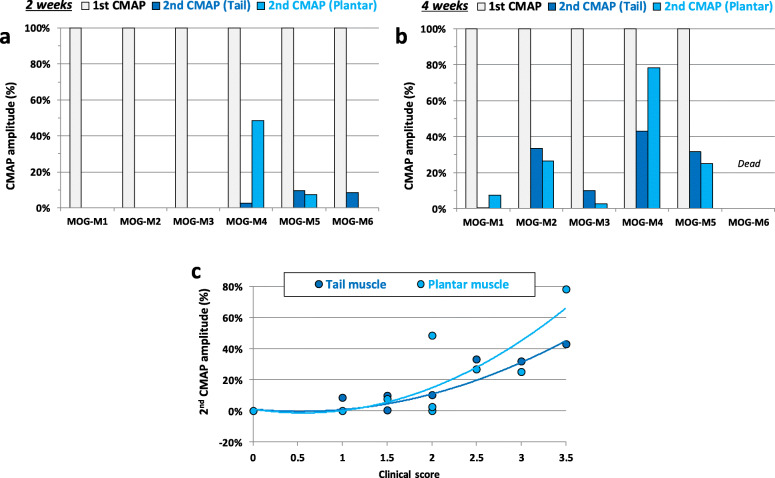
Expression of CMAP amplitude as a percentage of the first CMAP amplitude in individual EAE mice (MOG-M1 to MOG-6), 2 and 4 weeks after immunization (**a** and **b**), and relationship between the second CMAP amplitude and the EAE mouse clinical score (**c**)

Expressing the CMAP amplitude as a percentage of the first CMAP amplitude revealed a high inter-individual variability in the second CMAP amplitude (Fig. 9a–b), as already observed for the EAE mouse clinical scores (see Fig. 3). Indeed, establishing the relationship between the second CMAP amplitude and the EAE mouse clinical scores revealed a good correlation (*r*^2^ ≥ 0.677) between these two parameters (Fig. 9c). It is thus likely that the individual variability in both the presence and amplitude of the second CMAP reflected that of clinical scores.

In addition to the presence of a second CMAP in EAE mice, a significant decrease of the stimulation intensity necessary to generate a CMAP with an amplitude equal to 50% of its maximum value (*P* ≤ 0.038) was detected in MOG-mice, compared to CFA-animals, at any given time-point (Table 1, Supplementary Figures 1 and 2), as observed for the CNAP (see Fig. 5). However, the delay between the two CMAPs, measured as the time between their first peak amplitude, was constant, i.e., between 3.5 and 4 ms (see Fig. 8), and no significant difference of the time between the stimulus onset and the first peak amplitude of the first CMAP (*P* ≥ 0.292) was detected between EAE and control mice (Table 1, Supplementary Figures 1 and 2).

**Table 1:** Parameters (means ± S.D.) of the stimulus-response curve determined from tail and plantar muscles in n (numbers in parentheses) EAE (MOG) mice, compared to control (CFA) animals, 2 and 4 weeks after immunization

	2 weeks		4 weeks	
	CFA-mice (6)	MOG-mice (6)	CFA-mice (6)	MOG-mice (5)
Tail muscle				
CMAPmax (mV)	6.922 ± 0.773	3.893 ± 0.544*	5.992 ± 0.469	3.787 ± 0.511*
SI-50% (mA)	0.222 ± 0.014	0.179 ± 0.006*	0.224 ± 0.015	0.166 ± 0.013*
Slope	5.226 ± 0.451	3.258 ± 0.515*	4.977 ± 0.238	3.982 ± 0.112*
Latency (ms)	3.854 ± 0.067	3.703 ± 0.104	3.987 ± 0.130	3.818 ± 0.075
Plantar muscle				
CMAPmax (mV)	6.075 ± 0.650	3.204 ± 0.513**	5.635 ± 0.588	3.011 ± 0.522*
SI-50% (mA)	0.278 ± 0.023	0.174 ± 0.010**	0.320 ± 0.014	0.231 ± 0.013**
Slope	6.261 ± 0.650	3.559 ± 0.405*	5.756 ± 0.337	4.051 ± 0.206**
Latency (ms)	3.044 ± 0.201	3.625 ± 0.223	3.286 ± 0.147	3.346 ± 0.205

*CMAPmax* maximal CMAP amplitude, *SI*-*50%* stimulation intensity necessary to generate a CMAP with an amplitude equal to 50% of its maximum value, *Slope* slope of the stimulus-response curve, *Latency* time between the stimulus onset and the first peak amplitude of CMAP. *P ≤ 0.048 and **P ≤ 0.007

Additionally, and as already observed for the CNAP (see Fig. 5), a significant decrease of the maximal CMAP amplitude (*P* ≤ 0.039) and of the slope of the stimulus-response curve (*P* ≤ 0.048) was observed in MOG-mice, compared to CFA-animals, at any given time-point (Table 1, Supplementary Figures 1 and 2).

### Myelin sheath thickness of EAE and control mice

**Fig. 10:**
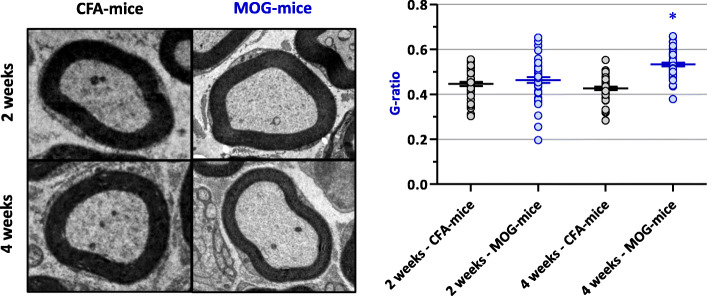
Peripheral nerve myelin sheath of EAE (MOG) mice, compared to control (CFA) animals, 2 and 4 weeks after immunization. Data represent the mean ± S.D. of 48 randomly chosen fibers per group, each constituted of 3 mice (i.e., 16 fibers per mouse). *P < 0.0001

To verify the alterations in the myelin contend induced in the EAE model, the morphology of the distal portion of the sciatic nerve was analyzed by transmission electron microscopy on the 2nd (peak of the disease) and 4th weeks after immunization. The thickness of the myelin sheath was quantified by calculating the g-ratio (i.e., the axon diameter divided by the fiber diameter). The results demonstrated intact fibers, with similar distribution of myelinic fibers of small and large diameters, non-myelinic fibers, and Schwann cell nuclei in the control group. No difference in myelin sheath thickness was observed between control and EAE mice, at the peak of the disease. In contrast, a decreased sciatic nerve myelin thickness, represented by an increase in the g-ratio, was observed in the EAE group, compared to control animals, in the 4th week after immunization (Fig. 10).

## Discussion

The results of this study can be summarized as follow: (i) a large inter-individual clinical score variability between EAE mice, (ii) a difference in body weight between EAE and control mice, (iii) modifications of the peripheral sensory nerve excitability parameters, and (iv) modifications of the neuromuscular excitability parameters.

The large inter-individual variability in clinical score between EAE mice may be attributed to differences in the susceptibility to EAE, as previously observed even in genetically identical animals [41]. Despite this variability in the intensity of the clinical score among the animals, all immunized mice developed some degrees of motor impairment characteristic of the disease, while none of the control animals showed this symptom.

We observed that EAE mice first lost and then progressively gained weight while control animals homogeneously gained weight, compared to initial body weight measurements performed at day 0. As a consequence, a difference in body weight between EAE and control mice, which is less marked as the number of weeks increased after immunization, was detected. These observations may be related to the fact that EAE mice had moving difficulties because of hind-limb paralysis and, therefore, did less exercise than control mice and that EAE animals showing a more than 2.0 clinical score were fed with highly nutritious food.

The results obtained from CNAP and CMAP recordings strongly suggest that the peripheral sensory nerve and neuromuscular excitability properties of EAE mice are markedly modified compared to those of control animals, 2 and 4 weeks after immunization. These modifications are not due to differences in body temperature since no significant change in this parameter was detected between EAE and control mice, whatever the number of weeks after immunization. They consist in a significant decrease of maximal CNAP and CMAP amplitudes, of the stimulation intensity necessary to generate a CNAP or CMAP with an amplitude equal to 50% of its maximum value, and of the slope of stimulus-response curves at any given time-point. In addition, a significant increase in the time between the stimulus onset and the first peak amplitude of CNAP was observed in MOG-mice compared to CFA-animals, at any given time-point, indicating a lower propagation velocity of the electrical transmission of the action potential in EAE than in control animals. In contrast, no significant difference of the time between the stimulus onset and the first peak amplitude of the first CMAP was detected between EAE and control mice. This strongly suggests that the chemical transmission of the action potential at the neuromuscular junction is not delayed. These electrophysiological abnormalities are similar to those previously reported in the ulnar and sural sensory nerves of some MS patients diagnosed according to the criteria of Poser Scale [28] and in superficial radial sensory axons of a few patients with relapsing-remitting MS [29]. They are also in accordance with the high frequency of electrophysiological abnormalities previously reported in the tibial motor nerve of a selected group of MS patients [28].

The significant decrease of the stimulation intensity necessary to generate a CNAP or a CMAP with an amplitude equal to 50% of its maximum value detected in MOG-mice, compared to CFA-animals, indicates sensory and motor nerve hyperexcitability in EAE mice compared to control animals. In agreement, membrane hyperexcitability of sensory neurons of dorsal root ganglia isolated from MOG_35-55_-induced EAE mice was previously reported [23]. Besides, the presence of a second CMAP in EAE mice may also express membrane hyperexcitability. However, the fact that the delay between the two CMAPs, measured as the time between their first peak amplitude, was constant strongly suggests that this second CMAP may also result from the activity of slow conducting motor nerve fibers, as expected if a demyelinating process occurs in the peripheral nervous system of EAE mice. Under these conditions, the first CMAP would be due to unaffected axons, whereas the second one would correspond to partially demyelinated axons. This is in agreement with the absence of detection of time difference between the stimulus onset and the first peak amplitude of the first CMAP in EAE mice, compared to control animals. Moreover, the recordings of multiple (at least 4) CNAP peaks may reflect not only the decrease of both the propagation velocity and the SI-50% parameter of CNAP but also various populations of sensory nerve fibers having different CNAP conduction speeds in EAE mice. This is expected if a demyelinating process occurred in the peripheral nervous system of EAE mice.

To support the occurrence of a peripheral demyelinating process, we investigated the alterations of sciatic nerve myelin contend by transmission electron microscopy, a methodology previously used as a parameter of peripheral myelin analysis for evaluation of neuroprotective therapies evaluation [42]. Although the widely expression of myelin peptides as MBP and MOG in the CNS is remarkable [43–46], these proteins are also expressed in the PNS [47–50], the thymus, and the spleen [51, 52]. These proteins can therefore be a target of autoantibodies attack, contributing to demyelination at peripheral level and, as a consequence, to peripheral sensory and motor alterations.

## Conclusion

In conclusion, the main modifications of the peripheral sensory nerve and neuromuscular excitability are (i) a membrane hyperexcitability likely related to membrane depolarization and (ii) the presence of slow conducting sensory and motor nerve fibers due to a demyelinating process occurring in the PNS of EAE mice. These modifications are of great interest since the loss of immunological self-tolerance to MOG in EAE animal model or in patients with MS is generally attributed to the restricted expression of this antigen in the immunologically privileged environment of the CNS.

## Supplementary information

**Additional file 1: Supplementary Figure 1.** Parameters of stimulus-response curves determined from tail muscle recordings in EAE (MOG) mice compared to control (CFA) animals. Supplementary **Figure 2.** Parameters of stimulus-response curves determined from plantar muscle recordings in EAE (MOG) mice compared to control (CFA) animals.

## Data Availability

All datasets [GENERATED/ANALYZED] for this study are included in the manuscript and the supplementary files.

## Abbreviations

ANOVA: Analysis of variance
ARRIVE: Animal Research: Reporting of In Vivo Experiments
CAP: Compound action potential
CAPmax: Maximal CAP amplitude
CFA: Complete Freund’s adjuvant
CMAP: Compound muscle action potential
CNAP: Compound nerve action potential
CNS: Central nervous system
EAE: Experimental autoimmune encephalomyelitis
IFA: Incomplete Freund’s adjuvant
K_V_ channel: Voltage-gated potassium channels
Latency: Time between the stimulus onset and the first peak amplitude of CAP
MBP: Myelin basic protein
MOG: Myelin oligodendrocyte glycoprotein
MS: Multiple sclerosis
Na_V_ channel: Voltage-gated sodium channel
PNS: Peripheral nervous system
SI-50%: Stimulation intensity necessary to generate a CAP with an amplitude equal to 50% of its maximum value
Slope: Slope of the stimulus-response curve
Th1: T-helper type 1 cell
Th17: T-helper type 17 cell
