# Supplementary material for: Alterations of peripheral nerve excitability in an experimental autoimmune encephalomyelitis mouse model for multiple sclerosis
Source: J Neuroinflammation. 2020 Sep 7;17:266. doi: 10.1186/s12974-020-01936-9 (PMC7487851; doi:10.1186/s12974-020-01936-9)
Supplement: Supplementary file 1 — Additional file 1: Supplementary Figure 1. Parameters of stimulus-response curves determined from tail muscle recordings in EAE (MOG) mice compared to control (CFA) animals. Supplementary Figure 2. Parameters of stimulus-response curves determined from plantar muscle recordings in EAE (MOG) mice compared to control (CFA) animals. [file 12974_2020_1936_MOESM1_ESM.pdf]

Supplementary Material

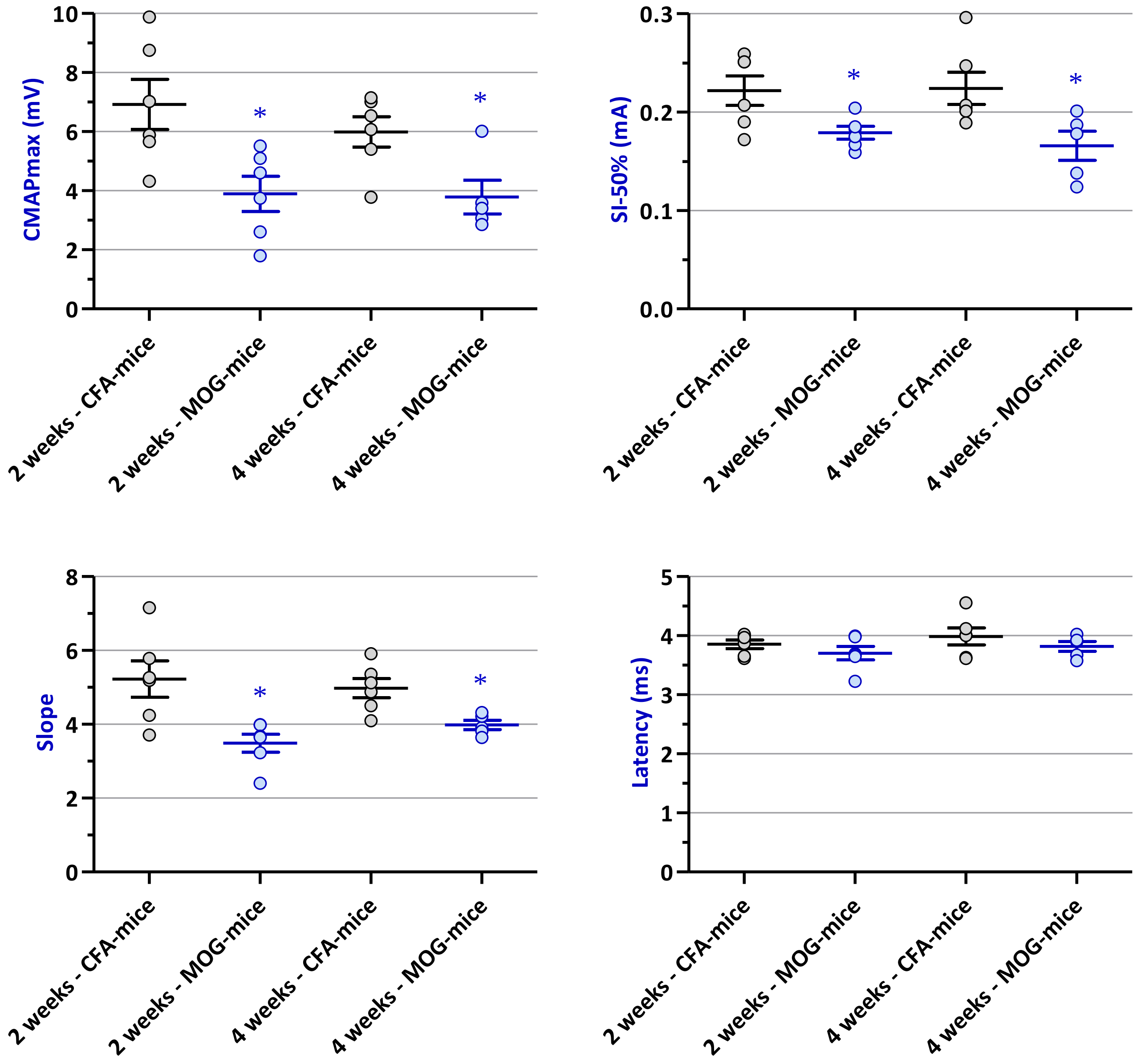

SUPPLEMENTARY FIGURE 1. Maximal CMAP amplitude (CMAPmax) recorded from the tail muscle, stimulation intensity necessary to generate a CMAP with an amplitude equal to 50% of its maximum value (SI-50%), slope of the stimulus-response curve (Slope) and time between the stimulus onset and the first peak amplitude of CMAP (Latency) in EAE (MOG) mice, compared to control (CFA) animals, 2 (n = 6) and 4 (n = 5-6) weeks after immunization. * : $P \leq 0.039$.

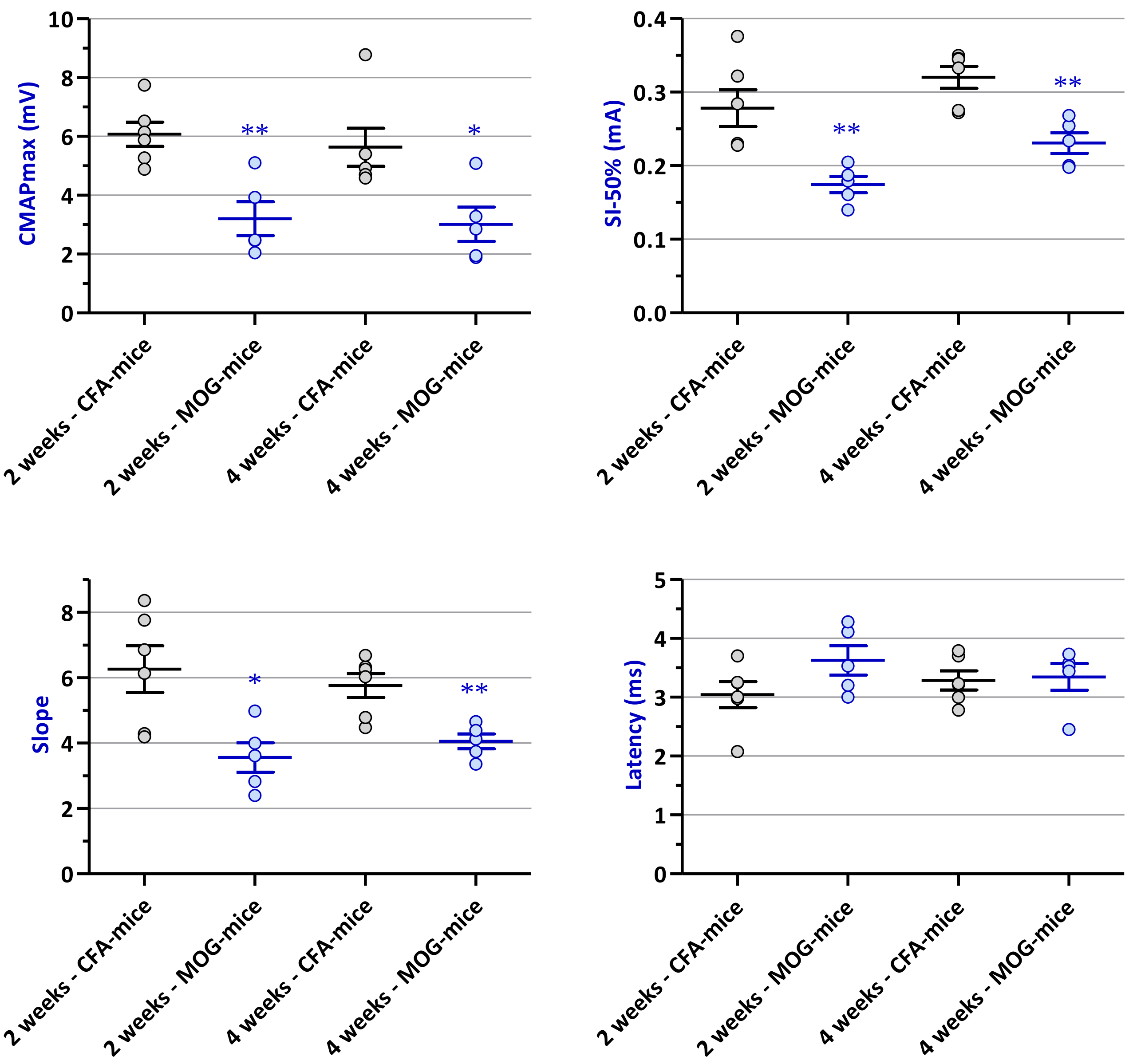

SUPPLEMENTARY FIGURE 2. Maximal CMAP amplitude (CMAPmax) recorded from the plantar muscle, stimulation intensity necessary to generate a CMAP with an amplitude equal to 50% of its maximum value (SI-50%), slope of the stimulus-response curve (Slope) and time between the stimulus onset and the first peak amplitude of CMAP (Latency) in EAE (MOG) mice, compared to control (CFA) animals, 2 (n = 5-6) and 4 (n = 5-6) weeks after immunization. * : P ≤ 0.048 and ** : P ≤ 0.007.
